# Evidence-based precision in TCM: A systematic review and Meta-Analysis of data mining for syndrome-specific acupoint combinations in acute asthma exacerbations

**DOI:** 10.1097/MD.0000000000050394

**Published:** 2026-08-28

**Authors:** Shili Yang, Yulong Yang, Yunlu Ding, Xiangrong Liu

**Affiliations:** a College of Nursing, Changchun University of Chinese Medicine, Changchun, China; b Department of Pharmacy, The Third Affiliated Hospital of Changchun University of Chinese Medicine, Changchun, China; c Experimental Center, Affiliated Hospital of Changchun University of Chinese Medicine, Changchun, China.

**Keywords:** acupoint application, bronchial asthma, frequent itemset analysis, latent structure analysis, syndrome differentiation and acupoint selection

## Abstract

**Background:**

The quest for personalized management of acute asthma exacerbations continues. Traditional Chinese Medicine (TCM) offers a rich repository of empirical knowledge, but its pattern-driven recommendations lack systematic, data-driven validation.

**Methods:**

We performed a systematic data mining analysis of TCM clinical literature on acupoint sticking therapy for acute asthma. Latent structure analysis and frequent itemset analysis were applied to 40 studies involving 2107 patients to objectively identify core syndrome patterns and their associated acupoint combinations.

**Results:**

We objectively delineated 2 predominant and clinically distinct syndrome patterns. For the pattern characterized by dyspnea with chills and clear sputum (analogous to “exterior-cold), the combination of Feishu (BL13), Dingchuan (EX-B1), and Shenshu (BL23) was most strongly associated. For the pattern with dyspnea, yellow sputum, and fever (analogous to “phlegm-heat), the combination shifted to Feishu (BL13), Dingchuan (EX-B1), and Dazhui (GV14).

**Conclusion:**

This computational study transforms qualitative TCM experience into quantifiable, syndrome-specific treatment rules. It provides an evidence-based framework for acupoint selection in acute asthma and demonstrates a novel methodology for extracting actionable clinical knowledge from historical texts. These findings offer a foundation for designing targeted clinical trials to validate personalized TCM interventions.

## 1. Introduction

Bronchial asthma is a prevalent chronic respiratory disease characterized by airway inflammation and hyper responsiveness, posing a significant global health burden.^[1]^ Acute exacerbations are critical events, driving healthcare utilization and constituting the primary cause of asthma-related mortality.^[2]^ Current first-line management relies on inhaled short-acting β_2_-agonists and corticosteroids for rapid symptom relief.^[3]^ While effective in the acute setting, this approach primarily targets symptom control and may not fully address the underlying disease heterogeneity or modify its long-term course. This therapeutic gap, alongside the growing emphasis on personalized medicine, has fueled interest in complementary strategies, including Traditional Chinese Medicine (TCM). Acupoint application (or sticking) therapy, a noninvasive external TCM modality with a history spanning over 2 millennia, is widely employed for acute asthma exacerbations. It is believed to promote bronchodilation, exert anti-inflammatory effects, and potentially reduce corticosteroid reliance.^[4]^ However, its efficacy is intrinsically tied to the TCM principle of syndrome differentiation, and the selection of acupoints remains largely empirical, lacking standardization due to variability in practitioners’ knowledge and clinical experience. Therefore, this study aims to address this gap by applying systematic data mining techniques to a body of TCM clinical literature. We therefore aimed to elucidate evidence-based, syndrome-specific acupoint selection patterns for acute asthma, and establish a reproducible analytical framework for transforming empirical medical knowledge into objective, testable clinical hypotheses.

## 2. Materials and methods

Reporting Guidelines: The design and reporting of this systematic data mining review followed the Preferred Reporting Items for Systematic Reviews and Meta-Analyses (2020 statement ^[5]^).

### 2.1. Data sources and search strategy

A systematic literature search was conducted across multiple electronic databases, including China National Knowledge Infrastructure, VIP, Wanfang, SinoMed, PubMed, Web of Science, Embase, and the Cochrane Library, from their inception to March 17, 2025. The search strategy employed a combination of Medical Subject Headings terms and free-text keywords related to 3 core concepts: asthma (e.g., “Bronchial asthma,” “Asthma bronchiale”); acupoint therapy (e.g., “Acupoint application,” “Transdermal acupoint administration”). The full search strategy for Embase (via Ovid) is provided as an example in Supplementary Material 1, Supplemental Digital Content 1.

### 2.2. Study selection criteria

Inclusion criteria were patients diagnosed with an acute exacerbation of bronchial asthma; intervention involving acupoint sticking therapy, either as a standalone treatment or in combination; and study types including randomized controlled trials, clinical observations, or case reports with detailed symptom and acupoint records.

Exclusion criteria were conference abstracts, reviews, meta-analyses, or animal studies; duplicate publications or studies with unavailable full–text; studies with incomplete baseline data or noncomparable groups; and studies that did not explicitly specify the acupoints used or the presenting symptoms.

### 2.3. Data extraction and standardization

Literature screening and management were performed using Note Express 3.4. Data from eligible studies were extracted into a standardized spreadsheet (Excel 2019) by 2 independent researchers. Discrepancies were resolved through consensus or consultation with a third senior TCM researcher. Extracted data included patient demographics, TCM syndrome patterns, symptoms, and acupoint prescriptions.

A rigorous standardization process was applied to ensure consistency. TCM syndrome and symptom terminology were standardized according to the Criteria for Diagnosis of Bronchial Asthma Syndromes in Traditional Chinese Medicine (2016 Edition)^[6]^ and the Clinical Terminology of Traditional Chinese Medicine (Syndrome Part) (GB/T 16751.2–2021).^[7]^ Acupoint nomenclature and localization followed the national standard Nomenclature and Location of Acupoints (GB/T 12346–2006).^[8]^ Implicit data (e.g., “cough with yellow sputum” implying both “cough” and “yellow sputum”) were explicitly coded by the 2 independent researchers, with high inter-rater agreement (Cohen’s kappa>0.85). The final dataset was structured as a binary matrix for computational analysis.

### 2.4. Statistical analysis: data mining techniques

Two complementary data mining approaches were employed to decode complex clinical patterns.

Latent structure analysis, a model-based technique, was used to identify unobservable (latent) TCM syndrome constructs from the observed symptom data. Analysis was performed using Lantern 5.0 software with the LTM-EAST algorithm. Models with varying numbers of latent variables were compared, and the optimal model was selected based on the lowest Bayesian Information Criterion score.^[9]^

Frequent itemset analysis: This rule-based technique was used to discover strong co-occurrence associations (rules) among symptoms, syndromes, and acupoints. The analysis was implemented using the Apriori algorithm in RStudio (v4.5.0). Key metrics were defined: Support (frequency of a rule in the dataset), Confidence (conditional probability of the consequent given the antecedent), and Lift (strength of association beyond random chance). To generate clinically meaningful and robust rules, minimum thresholds were set as follows: support ≥0.15 and confidence ≥0.8 for acupoint combination rules; confidence ≥ 0.9 for rules linking symptoms/syndromes to acupoints.^[10]^

### 2.5. Ethics approval

This study is a secondary analysis of previously published clinical literature. It did not involve direct interaction with human participants, the collection of new personal data, or the use of animal subjects. All data were extracted from publicly available published articles. Therefore, ethical approval from an institutional review board and informed consent were not required for this computational analysis.

## 3. Results

### 3.1. Literature search and study selection

**Figure 1.**
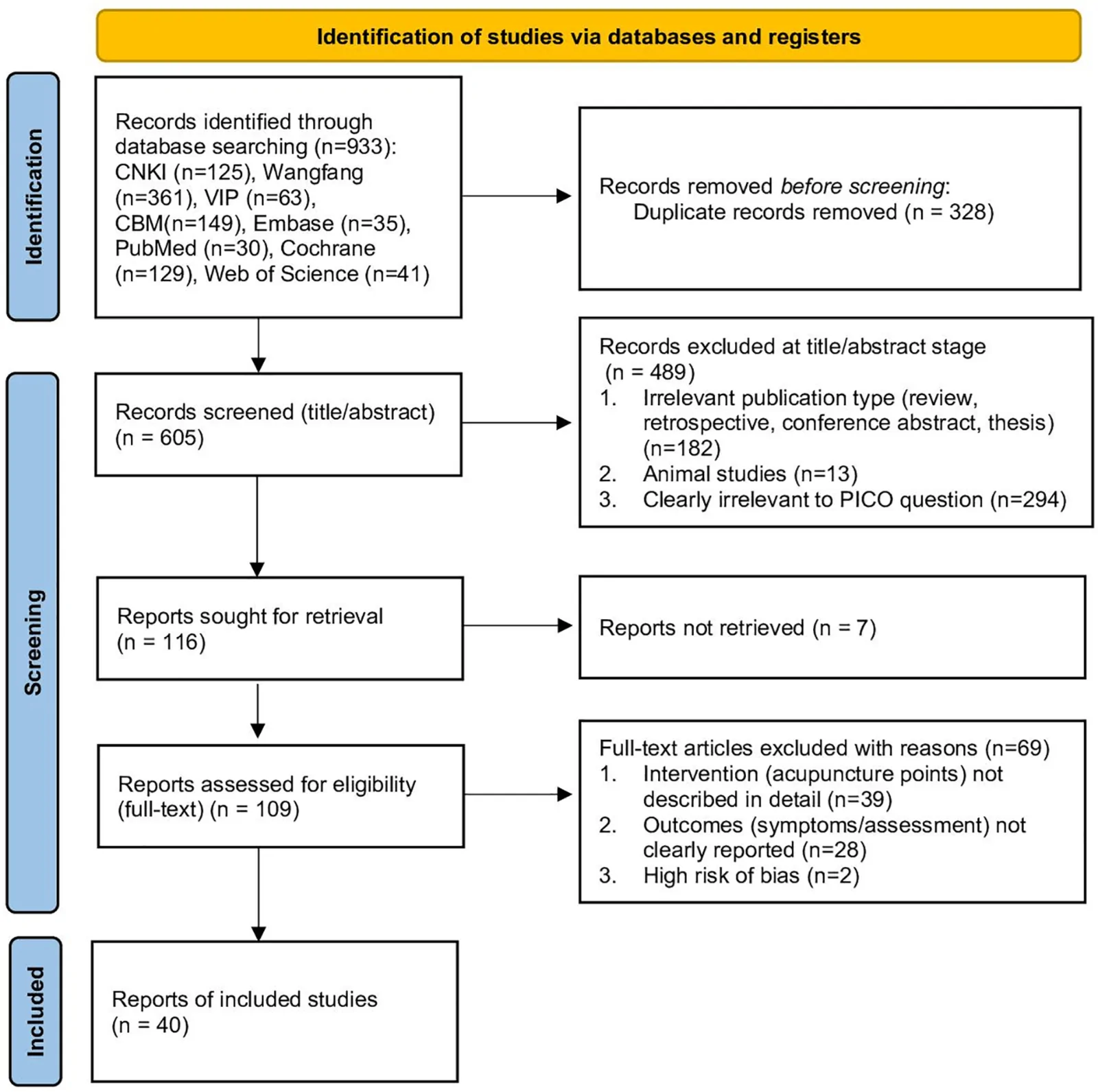
PRISMA Flowchart of study selection. The flowchart illustrates the identification, screening, eligibility, and inclusion process of studies. Initially, 933 records were identified through database searching. After removing duplicates, 605 records were screened. Following title/abstract screening, 116 full-text articles were assessed for eligibility. Ultimately, 40 studies were included in the final data mining analysis. CBM = Chinese Biomedical Literature Database, CNKI = China National Knowledge Infrastructure, PRISMA = Preferred Reporting Items for Systematic Reviews and Meta-Analyses.

The systematic search initially identified 933 records. After removal of duplicates, 605 unique articles were screened by title and abstract. Following this, 489 articles were excluded for not meeting the inclusion criteria, leaving 116 articles for full-text review. Ultimately, 40 studies^[11–50]^ were deemed eligible and included in the final data mining analysis. All included studies were clinical investigations. The study selection process is detailed in the flowchart (Fig. 1).

### 3.2. Patterns of acupoint selection in acute asthma exacerbation

#### 3.2.1. Frequency analysis of acupoints

The 40 included studies comprised a total of 2107 patients. Twenty-one distinct acupoints were utilized across these studies. Table 1 summarizes the ten most frequently applied acupoints (frequency ≥ 100). The three most prevalent acupoints were Feishu (BL13) (85.10%), Dingchuan (EX-B1) (84.86%), and Tiantu (CV22) (48.32%), indicating their foundational role in managing acute asthma attacks in clinical TCM practice.

**Table 1:** Analysis of high-frequency acupoints for acute bronchial asthma attacks (frequency ≥100).

Acupoint	Frequency (n)	Percentage (%)
Feishu (BL13)	1793	85.10
Dingchuan (EX-B1)	1788	84.86
Tiantu (CV22)	1018	48.32
Danzhong (CV17)	952	45.18
Dazhui (GV14)	682	32.37
Gaohuang (BL43)	462	21.93
Shenshu (BL23)	382	18.13
Pishu (BL20)	358	17.08
Geshu (BL17)	240	11.39
Yongquan (KI1)	115	5.46

#### 3.2.2. Frequent itemset analysis of acupoint prescriptions

Analysis of 44 distinct acupoint prescriptions using frequent itemset mining (support ≥ 0.15, confidence ≥ 0.8) yielded 13 significant association rules. The strongest association was observed between Feishu (BL13) and Dingchuan (EX-B1), with a support of 78.57% (i.e., this pair co-occurred in 78.57% of prescriptions) and a confidence of 0.90. The combination of Danzhong (CV17) and Feishu (BL13) also showed high confidence (1.00). These results suggest a core “back-chest” acupoint pairing strategy. The complete list of significant acupoint combination rules is presented in Table 2.

**Table 2:** Frequent itemset analysis of acupoint prescriptions for acute bronchial asthma attacks.

Antecedent	Consequent	Support (%)	Confidence (%)
Feishu (BL13)	Dingchuan (EX-B1)	78.57	0.9
Danzhong (CV17)	Feishu (BL13)	41.07	1
Dingchuan (EX-B1) + Danzhong (CV17)	Feishu (BL13)	35.71	1
Danzhong (CV17)	Dingchuan (EX-B1)	35.71	0.87
Tiantu (CV22)	Dingchuan (EX-B1)	33.93	0.86
Dazhui (GV14)	Dingchuan (EX-B1)	26.79	0.83
Feishu (BL13) + Tiantu (CV22)	Dingchuan (EX-B1)	25	0.88
Gaohuang (BL43)	Dingchuan (EX-B1)	23.21	1
Gaohuang (BL43)	Feishu (BL13)	23.21	1
Feishu (BL13) + Gaohuang (BL43)	Dingchuan (EX-B1)	23.21	1
Dingchuan (EX-B1)	Danzhong (CV17) + Tiantu (CV22)	17.86	0.9
Dingchuan (EX-B1)	Pishu (BL20) + Feishu (BL13)	16.07	1
Dingchuan (EX-B1)	Shenshu (BL23) + Feishu (BL13)	16.07	1

### 3.3. Associations among syndrome patterns, symptoms, and acupoints

#### 3.3.1. High-frequency symptoms

Fifty-seven symptoms were recorded across all cases. Dyspnea was the most prevalent symptom (96.68%), followed by chest tightness (85.29%) and cough (81.54%), reflecting the core clinical presentation of acute asthma. The top ten high-frequency symptoms are listed in Table 3.

**Table 3:** Analysis of high-frequency symptoms in acute bronchial asthma attacks.

Symptom	Frequency (n)	Percentage (%)
Dyspnea	2037	96.68
Chest tightness	1797	85.29
Cough	1718	81.54
Wheezing in the throat	1687	79.93
Shortness of breath	1314	62.36
Tachypnea	1214	57.62
Difficulty expectorating	920	43.66
White tongue coating	871	41.34
White sputum	843	40.01
Chills	812	38.54

#### 3.3.2. Latent structure model of symptoms

**Figure 2.**
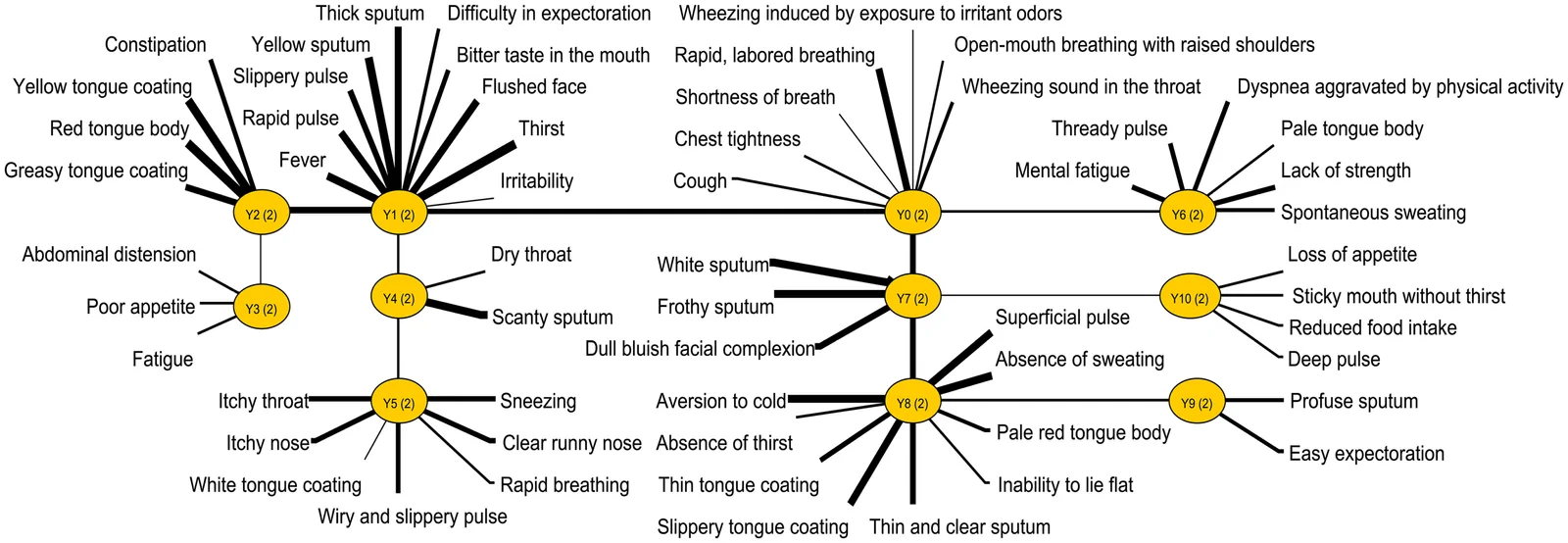
Latent structure model of symptoms in acute bronchial asthma attacks. The model identified 11 latent variables (Y0–Y10) representing 4 major TCM syndrome patterns: phlegm-heat obstructing the lung (Y1, Y2, Y3), wind-phlegm obstructing the lung (Y4, Y5), lung-spleen qi deficiency (Y6, Y10), and exterior-cold and interior fluid retention (Y7, Y8, Y9). Strong connections were observed between the primary symptom variable (Y0) and phlegm-heat and exterior-cold patterns, indicating these are the 2 predominant syndromes in acute asthma exacerbations.

Latent structure analysis was performed to uncover the underlying syndromic framework from the observed symptom data. The optimal model, achieving a cumulative coverage of 95% and a Bayesian Information Criterion score of −832.35, identified 11 latent variables (Y0–Y10), each comprising 2 latent classes. According to TCM theory, these latent variables were interpreted as representing 4 major syndrome patterns: phlegm-heat obstructing the lung (Y1, Y2, Y3), wind-phlegm obstructing the lung (Y4, Y5), lung-spleen qi deficiency (Y6, Y10), and exterior–cold and interior fluid retention (Y7, Y8, Y9). Strong connections were observed between the primary symptom variable (Y0) and the clusters representing phlegm-heat and exterior-cold patterns, suggesting these are the 2 predominant syndromes in acute exacerbations. The model structure is visualized in Figure 2.

#### 3.3.3. Symptom-acupoint association rules

Frequent itemset analysis focusing on symptom-acupoint associations (confidence ≥0.9, support ≥0.15) revealed 6 strong association rules. Feishu (BL13) was consistently selected when dyspnea, shortness of breathe, and chest tightness were the main symptoms. The addition of Dingchuan (EX-B1) was strongly associated when these core symptoms were accompanied by cough and wheezing in the throat. This indicates a hierarchical selection strategy based on symptom complexity. The detailed rules are shown in Table 4.

**Table 4:** Frequent itemset analysis of symptom-acupoint combinations in patients with acute bronchial asthma attacks.

Symptoms	Acupoints	Confidence (%)	Support (%)
Dyspnea + shortness of breath + chest tightness	Feishu (BL13)	1.00	59.65
Dyspnea + cough + shortness of breath + chest tightness	Feishu (BL13) + dingchuan (EX-B1)	1.00	52.63
Difficulty expectorating + wheezing in throat + chest tightness	Dingchuan (EX-B1)	1.00	42.11
Thick sputum + difficulty expectorating + thirst	Dazhui (GV14) + dingchuan (EX-B1) + Feishu (BL13)	1.00	21.05
Wheezing in throat + dry throat + Worsening dyspnea on exertion + Dyspnea + Scanty sputum	Feishu (BL13) + Danzhong (CV17) + Tiantu (CV22)	1.00	17.54
Chest tightness + Sneezing + Throat itch + Dyspnea	Feishu (BL13) + Shenshu (BL23)	1.00	17.54

#### 3.3.4. Syndrome-symptom-acupoint association rules

The most clinically informative findings emerged from the analysis integrating syndromes, symptoms, and acupoints (confidence ≥ 0.9, support ≥ 0.10). Two highly confident and specific rules were identified, corresponding to the 2 main syndrome patterns:

For exterior-cold and interior fluid retention syndrome, characterized by symptoms such as dyspnea, throat itch, sneezing, absence of thirst, thin tongue coating, and floating pulse, the associated acupoint combination was Feishu (BL13) + Dingchuan (EX-B1) + Shenshu (BL23).

For phlegm-heat obstructing the lung syndrome, characterized by thick yellow sputum, difficult expectoration, thirst, fever, flushed face, red tongue with yellow greasy coating, and rapid slippery pulse, the primary combination shifted to Dingchuan (EX-B1) + Feishu (BL13) + Dazhui (GV14).

These syndrome-specific, data-driven prescription rules are summarized in Table 5.

**Table 5:** Frequent itemset analysis of syndrome pattern-symptom-acupoint combinations in patients with acute bronchial asthma attacks.

Syndrome type	Symptoms	Acupoints	Confidence (%)	Support (%)
Exterior-cold and interior fluid retention syndrome	Dyspnea + throat itch + sneezing + absence of thirst + thin tongue coating + floating pulse	Feishu (BL13) + Dingchuan (EX-B1) + Shenshu (BL23)	1.00	33.33
Phlegm-heat obstructing the lung syndrome	Thick sputum + yellow sputum + difficulty expectorating + thirst + fever + flushed face + red tongue with yellow, greasy coating + rapid, slippery pulse	Dingchuan (EX-B1) + Feishu (BL13) + Dazhui (GV14)	1.00	32.14

## 4. Discussion

This study employed a novel, data-driven approach to systematically analyze the empirical knowledge contained within TCM literature for acute asthma management. By applying latent structure and frequent item set analysis to a corpus of clinical studies, we have systematically identified core syndrome patterns and their associated acupoint combinations. This approach moves beyond traditional narrative reviews, transforming qualitative experience into quantifiable, testable clinical hypotheses.

### 4.1. Decoding high-frequency acupoint combinations: a local and systemic strategy

Our analysis reveals a consistent clinical preference for specific acupoints during acute asthma exacerbations. The high-frequency selection of Feishu (BL13), Dingchuan (EX-B1), Tiantu (CV22), Danzhong (CV17), and Dazhui (GV14) aligns closely with the TCM principle of “Local Point Selection” and reflects a targeted anatomical and functional approach. Feishu (BL13), as the Back-Shu point of the lung, is fundamental for both alleviating symptoms (diffusing Lung Qi) and addressing the root cause (tonifying Lung Qi). Dingchuan (EX-B1), an empirical extraordinary point, acts as a critical “asthma-relief” point for rapid symptom control. The high-support pairing of Feishu (BL13) and Dingchuan (EX-B1) embodies the synergistic principle of “simultaneously treating the root and the manifestation.” This core duo, when integrated with points like Danzhong (CV17, the Sea of Qi) and Dazhui (GV14, a major Yang confluence), forms a multidimensional therapeutic network. This network spatially spans the back (Dingchuan, Feishu), anterior chest (Danzhong), and upper spine (Dazhui), enabling a coordinated attack on multiple pathological facets: bronchospasm, phlegm congestion, and disrupted Qi dynamics. This finding provides a data-backed rationale for commonly observed clinical prescriptions.

### 4.2. Syndrome-symptom-acupoint correlations: towards precision TCM

The most significant contribution of this study is the objective delineation of syndrome-specific acupoint protocols, directly supporting the core TCM tenet of “treatment based on pattern differentiation.”

For the Exterior-Cold and Interior Fluid Retention pattern, the data strongly associate the combination Feishu (BL13) + Dingchuan (EX-B1) + Shenshu (BL23). Shenshu (BL23), the Back-Shu point of the kidney, is key here. Its inclusion shifts the therapeutic strategy from merely addressing the lung (the manifestation) to reinforcing the kidney’s function of grasping Qi (the root), a principle emphasized in classical texts like Jingyue’s Complete Compendium for treating dyspnea.^[51]^ This combination creates a strategy of “warming the exterior, directing Qi downward, and reinforcing the root.”

For the Phlegm-Heat Obstructing the Lung pattern, the protocol shifts to Dingchuan (EX-B1) + Feishu (BL13) + Dazhui (GV14). The substitution of Dazhui (GV14) for Shenshu (BL23) is critical. Dazhui, a powerful point for clearing heat and releasing the exterior, directly counters the “heat” component of this syndrome. This combination establishes a “clear heat, transform phlegm, free the airway” pathway, which aligns with modern understanding of managing inflammatory (heat) and obstructive (phlegm) components in acute asthma.^[52]^

These data-mined rules offer a structured framework for clinicians, potentially reducing variability in acupoint selection and facilitating more reproducible, syndrome-targeted interventions.

### 4.3. Methodological implications and future directions

This study demonstrates the utility of computational text mining as a bridge between traditional empirical knowledge and modern evidence-based research. The methodology- systematic literature aggregation, rigorous data standardization, and unbiased pattern recognition- provides a replicable framework for knowledge discovery in other domains of complementary and integrative medicine.

The specific acupoint combinations identified, particularly the 2 syndrome-specific core sets, are no longer just empirical suggestions but priority candidates for focused clinical validation. We propose that these combinations serve as the basis for future rigorously designed randomized controlled trials. Such trials should compare the efficacy of these data-derived, syndrome-matched protocols against standard care or nonspecific acupoint application.

### 4.4. Limitations

This study has several limitations that should be acknowledged. First, the analyzed literature predominantly consists of Chinese-language studies, which may introduce language and publication bias. Second, the data extraction and symptom/syndrome standardization, despite high inter-rater agreement, inherently involve interpretive judgment based on TCM theory. Third, the included studies varied in design and quality; while data mining is robust to heterogeneity, it cannot compensate for potential biases in the source material. Finally, this is a computational analysis of textual data; the clinical efficacy of the identified combinations must be prospectively validated.

## 5. Conclusion

In conclusion, through a dual data mining approach, this study has objectively identified exterior-cold and interior fluid retention and phlegm-heat obstructing the lung as the 2 predominant TCM syndrome patterns in acute bronchial asthma exacerbations. For each pattern, a core, syndrome-specific acupoint combination was derived: Feishu-Dingchuan-Shenshu for the former, and Dingchuan-Feishu-Dazhui for the latter. This work translates historical TCM experience into a structured, evidence-informed framework for personalized acupoint selection. It provides an immediate, data-grounded reference for clinical practice. More importantly, it pinpoints specific, syndrome-matched acupoint combinations- Feishu-Dingchuan-Shenshu and Dingchuan-Feishu-Dazhui- as prime candidates for validation in pragmatic randomized controlled trials, thereby paving the way for integrating personalized TCM strategies into contemporary asthma management protocols.

## Acknowledgments

During the preparation of this manuscript, the authors used DeepSeek for translation and language polishing from Chinese to English. The authors have thoroughly reviewed, edited, and take full responsibility for the final content of this publication.

## Author contributions

**Methodology:** Shili Yang, Yunlu Ding.

**Data curation:** Yulong Yang, Yunlu Ding.

**Investigation:** Yulong Yang.

**Validation:** Yunlu Ding, Xiangrong Liu.

**Funding acquisition:** Xiangrong Liu.

**Resources:** Xiangrong Liu.

**Supervision:** Xiangrong Liu.

**Writing – review & editing:** Xiangrong Liu.

**Writing – original draft:** Shili Yang.


