# Supplementary material for: Evidence-based precision in TCM: A systematic review and Meta-Analysis of data mining for syndrome-specific acupoint combinations in acute asthma exacerbations
Source: Medicine (Baltimore). 2026 Aug 28;105(35):e50394. doi: 10.1097/MD.0000000000050394 (PMC13529163; doi:10.1097/MD.0000000000050394)
Supplement: Supplementary file 1 [file medi-105-e50394-s001.docx]

**Supplementary Material 1: Detailed Search Strategies**

**Database:** Embase
**Platform:** [embase.com](https://embase.com/)
**Date of Search:** March 17, 2025

**Search Strategy:**

| # | Searches | Results |
| --- | --- | --- |
| #1 | 'asthma'/exp | 338,232 |
| #2 | 'asthma bronchiale':ab,kw,ti OR 'asthma pulmonale':ab,kw,ti OR 'asthma, bronchial':ab,kw,ti OR 'asthmatic':ab,kw,ti OR 'asthmatic subject':ab,kw,ti OR 'bronchial asthma':ab,kw,ti OR 'bronchus asthma':ab,kw,ti OR 'chronic asthma':ab,kw,ti OR 'lung allergy':ab,kw,ti OR 'asthma':ab,kw,ti | 290,823 |
| #3 | #1 OR #2 | 380,542 |
| #4 | 'acupoint application':ab,kw,ti OR 'point application':ab,kw,ti OR 'acupuncture application therapy':ab,kw,ti OR 'therapy of point application':ab,kw,ti | 284 |
| #5 | #3 AND #4 | 35 |

**Chinese Database Search Strategies**

**Date of Search:** March 17, 2025

**SinoMed (Chinese Biomedical Literature Database):**

| # | Search Expression | Results |
| --- | --- | --- |
| #1 | "asthma" /exp | 78,659 |
| #2 | "bronchial asthma":TI,AB,KW OR "asthma syndrome":TI,AB,KW OR "asthma pattern":TI,AB,KW OR "dyspnea syndrome":TI,AB,KW | 116,451 |
| #3 | #1 OR #2 | 116,451 |
| #4 | "acute phase":TI,AB,KW OR "acute exacerbation":TI,AB,KW OR "acute attack stage":TI,AB,KW OR "attack stage":TI,AB,KW | 84,986 |
| #5 | "acupoint application therapy" /exp | 4,496 |
| #6 | "acupoint application":TI,AB,KW OR "acupoint patching":TI,AB,KW OR "transdermal acupoint administration":TI,AB,KW OR "meridian point administration":TI,AB,KW OR "herbal patch":TI,AB,KW OR "herbal application":TI,AB,KW OR "acupoint herbal patching":TI,AB,KW OR "acupoint herbal application":TI,AB,KW OR "external herbal application":TI,AB,KW | 32,186 |
| #7 | #5 OR #6 | 32,490 |
| #8 | #3 AND #4 AND #7 | 149 |

**Notes:**

- The search strategies employed a combination of controlled vocabulary (Emtree/MeSH terms) and free-text keywords related to three core concepts: asthma, acute phase, and acupoint therapy.
- Boolean operators: OR was used to combine synonyms within each concept; AND was used to link the three core concepts.
- Limits: Results were limited to human studies and clinical research types; animal studies, conference abstracts, reviews, letters, and notes were excluded.
- The Chinese database search was conducted using SinoMed (Chinese Biomedical Literature Database). Similar search strategies were applied to CNKI, Wanfang, and VIP databases with appropriate syntax adjustments for each platform.
